# MRI Assessment of Cardiomyopathy Induced by β1-Adrenoreceptor Autoantibodies and Protection Through β3-Adrenoreceptor Overexpression

**DOI:** 10.1038/srep43951

**Published:** 2017-03-09

**Authors:** Laetitia Vanhoutte, Céline Guilbaud, Ruben Martherus, Caroline Bouzin, Bernard Gallez, Chantal Dessy, Jean-Luc Balligand, Stéphane Moniotte, Olivier Feron

**Affiliations:** 1 Pole of Pharmacology and Therapeutics (FATH), Institut de Recherche Expérimentale et Clinique (IREC), Université catholique de Louvain, Brussels, Belgium; 2 Department of Paediatric Cardiology, Cliniques universitaires St Luc, Université catholique de Louvain, Brussels, Belgium; 3 Biomedical Magnetic Resonance Unit (REMA), Louvain Drug Research Institute (LDRI), Université catholique de Louvain, Brussels, Belgium

## Abstract

The cardiopathogenic role of autoantibodies (aabs) directed against β1-adrenoreceptors (β1-AR) is well established. In mouse models, they cause progressive dilated cardiomyopathy (DCM) whose characterization with echocardiography requires prolonged protocols with numerous animals, complicating the evaluation of new treatments. Here, we report on the characterization of β1-aabs-induced DCM in mice using 11.7T MRI. C57BL/6J mice (n = 10 per group) were immunized against the β1-AR and left ventricular (LV) systolic function was assessed at 10, 18 and 27 weeks. Increase in LV mass/tibial length ratio was detected as the first modification at 10 weeks together with dilation of cavities, thereby outperforming echocardiography. Significant impairment in diastolic index was also observed in immunized animals before the onset of systolic dysfunction. Morphometric and histological measurements confirmed these observations. The same protocol performed on β3-AR-overexpressing mice and wild-type littermates (n = 8–12 per group) showed that transgenic animals were protected with reduced LV/TL ratio compared to wild-type animals and maintenance of the diastolic index. This study demonstrates that MRI allows a precocious detection of the subtle myocardial dysfunction induced by β1-aabs and that β3-AR stimulation blunts the development of β1-aabs-induced DCM, thereby paving the way for the use of β3AR-stimulating drugs to treat this autoimmune cardiomyopathy.

Because of its high prevalence, morbidity and mortality, heart failure remains a major health problem. Among the mechanisms leading to this condition, the detrimental role played by autoantibodies targeting the β1-adrenergic receptor (β1-aabs) has been established in the last two decades. Clinical studies have shown that the prevalence of β1-aabs is associated with dilated cardiomyopathy (DCM)^1^,^2^,^3^, ischemic cardiomyopathy^4^ and Chagas’ disease^5^, with – overall – a more frequent occurrence of poor LV function^4^, ventricular arrhythmias^6^, sudden cardiac death^6^,^7^ and mortality^8^. By contrast, prevalence is apparently lower in patients with heart failure due to valvular or hypertensive diseases^9^. These gross statistics require further clarification in order to better understand the time course of appearance and exact roles of these antibodies in patients suffering from cardiac diseases of various etiologies. A multicentric prospective and retrospective study is currently conducted in patients suffering from myocarditis, ischemic and hypertensive heart diseases^10^.

Evidence has accumulated from animal studies confirming the direct pathogenicity of β1-aabs. A highly antigenic fragment containing B and T cell epitopes has been identified on the second extracellular loop (β1-EC_II_) of the β1-adrenoreceptor (β1-AR)^11^,^12^,^13^. Studies on animal models have established the development of cardiac dysfunction after the active immunization against this antigen (indirect evidence)^14^,^15^,^16^ and assessed the possibility of generating cardiac impairment after transfer of homologous pathogenic antibodies to healthy animals (direct evidence)^17^. The two main hypotheses raised to explain the development of such antibodies include homologies between the receptor and microbial determinants or the exposition of potentially antigenic components of the cardiomyocytes following cardiac damages^18^.

The functional effects of β1-aabs at the cellular and molecular levels are incompletely understood. Preliminary results suggest that they act as allosteric agonists^19^, enabling dimerization and stabilization of β1-AR in its active conformation^20^, subsequently promoting sustained downstream signaling in a manner distinct from the natural ligand^21^,^22^. This leads to cardiomyocyte apoptosis^23^, enhanced contractility and prolonged action potential duration^24^. During progression of aabs-induced cardiomyopathy, increased levels of GRK2 have also been documented in several rodent studies^16^,^25^,^26^, suggesting desensitization and down-regulation of β1-AR. Some therapies, including the use of apheresis^27^, aptamers^28^, peptides competing with the receptor epitope^29^,^30^ and conventional beta-blockers^30^,^31^ have proven to be useful in order to partially prevent the development of cardiac dysfunction due to β1-aabs.

The β3-adrenoreceptor (β3-AR) has recently emerged has a potential therapeutic target in the presence of an excessive β-adrenergic stimulation on the heart, as it mediates a countervailing influence to β1 and β2-AR activation^32^ and is resistant to homologous desensitization^33^. Moreover β3-ARs are upregulated under various conditions of adrenergic overstimulation^34^,^35^,^36^, arguing in favor of a potential influence of these receptors on chronic remodeling. Our group has recently demonstrated that transgenic mice with cardiac-specific overexpression of β3-AR were protected from hypertrophic and fibrotic remodeling due to neurohormonal stimulation^37^. Whether β3 stimulation may prevent or correct autoimmune DCM is however unknown.

Up to now, the anatomical characterization of the cardiomyopathy induced by β1-aabs in animals has mostly been performed using echocardiography. However, cardiac dysfunction appears slowly and frequently starts with subtle diastolic dysfunction and elevated filling pressures. With the limited accuracy and reproducibility of echocardiography, prolonged protocols with numerous animals are needed before identifying early modifications in immunized individuals. The recent development of ultra-high field MRI (UHF MRI) has brought new perspectives in cardiovascular imaging of small animal models, due to its higher accuracy and reproducibility^38^ and the numerous sequences available. Here, we studied the evolution of systolic and diastolic LV function of mice submitted to β1-AR immunization with an 11.7T MRI scanner to evaluate whether this technique could permit earlier detection of remodeling induced by aabs. We also aimed to study whether β3-AR overexpressing mice could be protected from cardiac remodeling induced by chronic exposure to β1-aabs.

## Results

### Mouse model of β1AR aabs-induced cardiomyopathy

**Figure 1:**
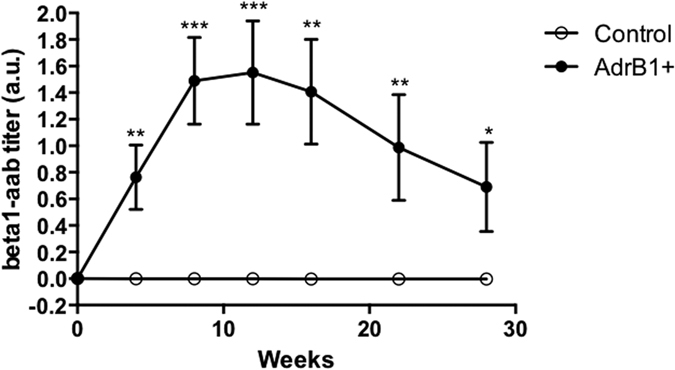
Titer of antibodies against β1AR-ECII determined by ELISA throughout the study period, in sera of control (CTL, n = 10) and β1-immunized (AdrB1+, n = 7–10) mice. Data are represented as mean+/−SEM, and normalized to basal optical density obtained before injection, *P ≤ 0.0.05; **P ≤ 0.0.01; ***P ≤ 0.0.001*vs* control.

We actively immunized C57Bl/6J mice against the β1-AR through monthly subcutaneous injections of a peptide corresponding to the second extracellular loop of the receptor, for a total of 28 weeks. In parallel, control animals were s.c. administered the vehicle solution. Immunized mice showed a gradual increase of circulating antibodies directed against β_1_AR-EC_II_, reaching a peak after 2 booster doses, whereas β_1_-aabs remained undetectable in control mice (Fig. 1). 3 mice died in the immunized group within the first 12 weeks of protocol. Deaths occurred in animals presenting the most rapid increase in antibody titers, suggesting the occurrence of aabs-induced lethal arrhythmias^39^,^40^. No alterations in body weight, food intake and behavior were observed in surviving mice.

### MRI cardiac function evaluation upon induction of β1AR aabs

**Figure 2:**
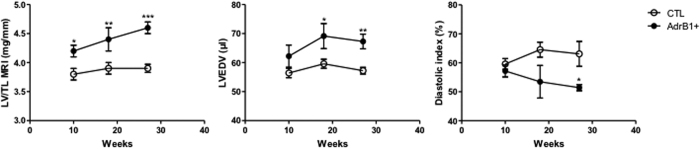
MRI follow-up in β1-immunized (n = 7–9) and control mice (n = 10). The panels depict the time course of the following parameters: LV mass/tibial length ratio (LV/TL), end-diastolic volume (EDV) and diastolic index. Error bars indicate SEM. *P ≤ 0.0.05; **P ≤ 0.0.01; ***P ≤ 0.0.001; ****P ≤ 0.0.0001 *vs* control.

After 10 weeks of follow-up, mice immunized against the β_1_AR-EC_II_ already showed a statistically significant increase in the LV/TL ratio compared to control mice (4.2 ± 0.1 *vs* 3.8 ± 0.1 mg/mm, p < 0.05) (Fig. 2, left panel and Table 1). Over time, a continuous increase of this parameter was observed in the β_1_-immunized group, accompanied by signs of progressive dilation of LV (Fig. 2, left panel and Table 1). These MRI data for LV mass and LV/TL were confirmed by *ex vivo* measurements at 28 weeks (Table 1).

**Figure 3:**
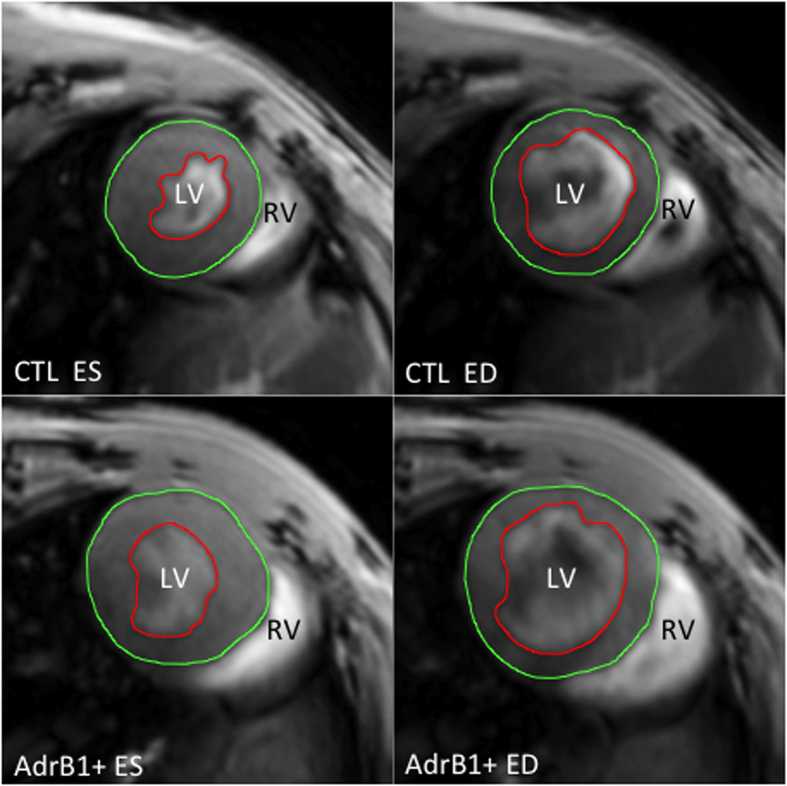
Representative MRI short-axis views of mouse hearts after 27 weeks of evolution in end-diastolic (ED) and end-systolic (ES) phases, with endocardial (red) and epicardial (green) contours, showing left ventricular dilation in β1-immunized (AdrB1+) vs control mice. LV = left ventricle, RV = right ventricle.

A statistically significant difference for EDV was noticeable after 18 weeks between treated and control mice (69.1 ± 4.3 *vs* 59.6 ± 1.5 μl, p < 0.05) (Fig. 2, middle panel and Table 1), and after 27 weeks for ESV (29.0 ± 4.0 *vs* 22.0 ± 1.5 μl, p < 0.05) (Table 1). No difference was observed between the two groups for end-systolic septum thickness. Figure 3 shows representative short-axis images in β_1_-immunized and control mice at the end of the protocol.

Analysis of the diastolic index showed an impaired diastolic function in the treated group from 18 weeks that become significantly altered at 27 weeks (51.4 ± 1.1 *vs* 63.1 ± 4.3%, P < 0.05) (Fig. 2, right panel and Table 1). By contrast, systolic function assessed both by EF and systolic index remained within normal values in the immunized group (Table 1).

### Cardiac histology and gene expression upon induction of β1AR aabs

**Figure 4:**
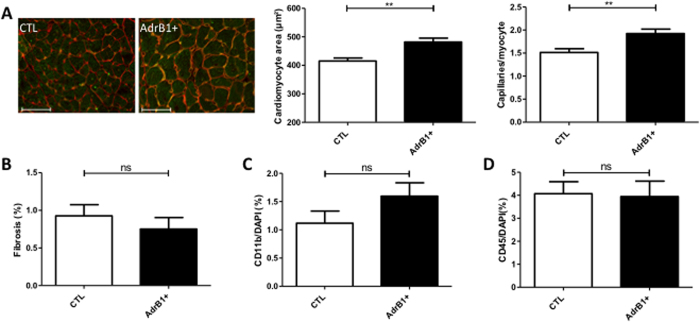
Histology of mouse hearts. (**A**) Left panel: representative cross-sections of hearts from control and β1-immunized (AdrB1+) mice stained with isolectin B4 (green) and wheat germ agglutinin (red). Scale bar, 50 μm. Right panel: columns indicate the respective myocyte area and capillary density from same sections in each group, showing cardiomyocyte hypertrophy compensated by higher capillary density in hearts from immunized mice. (**B**) Quantification of collagen infiltration with sirius red staining. (**C,D**) Quantification of inflammatory infiltration with CD11b and CD45 stainings. *P ≤ 0.0.05; **P ≤ 0.0.01 *vs* control. n = 7–10 mice per group.

Histomorphometric analysis of cardiomyocyte transverse area confirmed significantly larger myocytes with increased vascular density in β_1_-immunized mice (p < 0.05) (Fig. 4A). No differences in collagen (Sirius red labeling) (Fig. 4B) or inflammatory infiltration (CD45/CD11b staining) (Fig. 4C and D) were observed between the two groups.

We also performed qPCR analyses to identify changes in mRNA expression from corresponding cardiac tissues (Table 2). mRNA expression of βAR-1 and -2 (ADRB 1–2) remained unchanged between control and β_1_-immunized group while a two-fold increase in βAR-3 gene expression (ADRB3) was observed in the immunized group (although not reaching statistical significance, Table 2). No re-expression of the fetal gene program was found based on the levels of myosin heavy chains (MYH6 and MYH7 coding for the alpha and beta isoform, respectively) and natriuretic peptides (ANP and BNP for atrial and brain forms, respectively); a trend toward an increase in the BNP gene expression was however observed in β_1_-immunized mice (p = 0.07, Table 2).

### β3TG mice are protected from β1AR aabs-induced cardiomyopathy

We tested whether the overexpression of the β3-AR could protect from developing β1-aabs-induced DCM by applying the protocol of immunization previously described to 12 transgenic (heterozygous) mice overexpressing the β3-AR (TG) and 12 wild-type littermates (WT). 5 mice died in each group upon active immunization against β_1_AR-EC_II_ before the end of the protocol.

**Figure 5:**
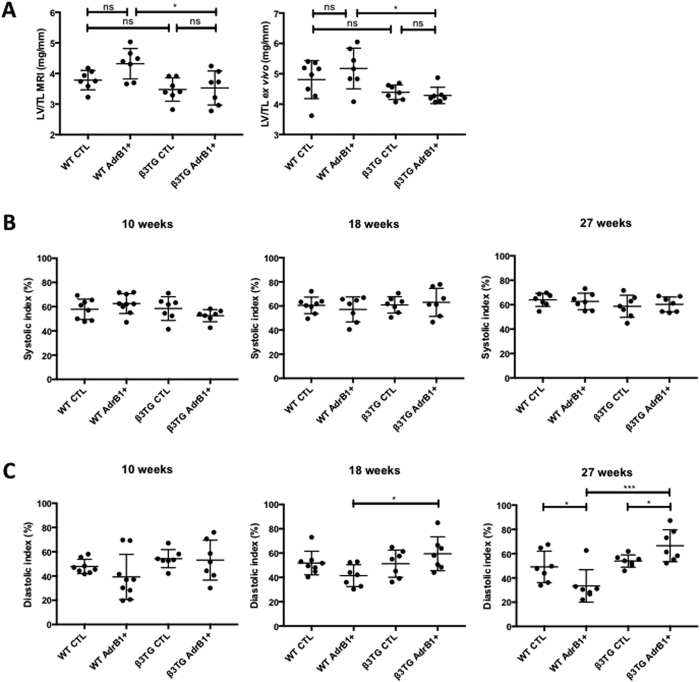
(**A**) MRI LV/TL (left) and *ex vivo* LV/TL (right) ratios at 27 and 28 weeks, respectively, in transgenic mice overexpressing the beta3 adrenergic receptor and immunized (TG AdrB1+) or not (TG CTL) against AdrB1-ECII, and their wild-type littermates with (WT AdrB1+) or without (WT CTL) immunization. Systolic **(B)** and diastolic **(C)** indexes determined in the same animals after 10, 18 and 27 weeks, *P ≤ 0.0.05; **P ≤ 0.0.01 as indicated.

Cardiac MRI measurements after 27 weeks showed statistically significant differences between the two immunized groups: LV/TL ratio remained unaltered in immunized β_3_-overexpressing mice, with a value of 3.5 ± 0.2 mm/mg (*vs.* 4.3 ± 0.2 mm/mg in immunized wild-type animals, p < 0.05) (Fig. 5A, left panel). These data were confirmed by *ex vivo* LV/TL measurements after sacrifice of the animals (Fig. 5A, right panel). Interestingly, while determination of systolic indexes did not reveal any difference between mouse groups (Fig. 5B), evaluation of diastolic indexes reinforced the concept of β3-AR-mediated protection. First, significant differences between the two immunized groups were observed as soon as 18 weeks (Fig. 5C). Second, while LV/TL ratio measurements only revealed a trend towards an increase in control littermates of transgenic animals (see two first sets of points in Fig. 5A graphs), diastolic indexes revealed a statistically significant reduction after 27 weeks, confirming the earliness of diastolic dysfunction in this DCM model (Fig. 5C). Other MRI measurements were not altered when compared to control groups after 27 weeks of follow-up (not shown).

## Discussion

The major findings of this study are related to the application of UHF MRI technology to study the early development of β1aabs-driven autoimmune cardiac dysfunction and to the identification of β3-AR as key actor of a counterbalancing pathway preventing the occurrence of DCM in this model.

Previous studies using echocardiography have reported the first anatomical changes at rest in β_1_-immunized BalbC^25^ and C57BL/6J mice^26^ at 25 weeks. In our study with C57BL/6J mice immunized against the β1-AR, using UHF MRI, we were able to show early aabs-induced cardiomyopathic changes after only 10 weeks. LV hypertrophy (increase in LV/TL ratio) was detected before transition to the complete phenotype of DCM. Furthermore, we applied to our UHF MRI study a recently described method based on the concepts of echocardiographic color kinesis^41^,^42^ in order to derive systolic and diastolic indexes in our animals. This led us to document that diastolic dysfunction was detectable before the occurrence of systolic dysfunction. These results open new perspectives for clinical investigations in patients carrying β1-aabs. Indeed, several methods (i.e. echographic speckle tracking and MR tagging ref. 43) are now available besides the classical echocardiographic mitral inflow color Doppler (E/A) measurements in order to thoroughly investigate diastolic function in patients. If these data are confirmed by further clinical studies, appearance of diastolic dysfunction in β1-aabs-positive patients could be used as an early marker of evolution to heart failure, requiring an intensification of treatments.

Besides the above technological benefits of UHF MRI applied to autoimmune DCM, we showed that transgenic mice harboring a cardiac-specific human β3-AR transgene were protected from myocardial dysfunction induced by β1-aabs. Although, unlike in C57BL/6J mice, only a trend to a cardiac dilation (ie, increased LV/TL ratio) was detectable in the immunized WT group (*vs.* non-immunized mice) at the end of our study, a significant reduction in the diastolic index confirmed the development of β1aabs-mediated cardiac alterations in these mice. Different genetic backgrounds are likely to account for the LV/TL ratio discrepancy but this observation also emphasize the earliness of the diastolic dysfunction in this model of autoimmune DCM. Importantly, in β3-AR overexpressing mice, the diastolic index was not decreased in response to immunization and was actually even slightly increased. Altogether, these data indicate a significant protective effect of β3-AR overexpression against the development of heart damage induced by β1-aabs (*vs.* immunized WT mice) and thereby open new therapeutic perspectives in the treatment of autoimmune DCM.

As β3-AR have functionally opposite effects to β1-AR and β2-AR on cardiac muscle and are more resistant than β1-AR and β2-AR to homologous desensitization^33^, they represent attractive candidates for efficient pharmacological modulation in the diseased heart^44^. Recent data published by our team have already shown that β3TG mice were protected against early cardiac dysfunction induced by neurohormone infusion (i.e. angiotensin II or isoproterenol)^37^. The present study confirms the potential beneficial effects of β3-adrenergic stimulation to also prevent the development of autoimmune DCM. In addition to the attenuation of β1-adrenergic inotropic responses, the nitric oxide production consecutive to a β3-AR stimulation of microvascular endothelial cells^45^ and cardiac myocytes^37^ may help to maintain a normal left ventricular diastolic function during the early stages of cardiac dysfunction; improving diastolic relaxation can indeed exert a beneficial hemodynamic effect through the maintenance of the Frank-Starling response^46^. The latter property seems even more relevant for the treatment of β1-aabs-induced cardiomyopathy, in the light of the early diastolic dysfunction reported in mice immunized against β1-EC_II_.

A limitation in our study is the use of transgenic β3 AR-overexpressing mice which *per se* means that this pathway is continuously influencing the cardiac phenotype of these animals and may thus prevent dysfunction from the earliest stages of its development. Still, these results pave the way for the evaluation of *bona fide* β3-AR agonists or the preferred use of β-blockers endowed with such β3-AR stimulatory activity in the treatment of autoimmune β1-aabs-induced DCM. Nebivolol represents a third generation selective β1-AR antagonist with ancillary metabolic effects involving a β3-AR stimulation. Nebivolol could thus bring an increment of efficacy *vs.* conventional beta-blockers whose usefulness has already been demonstrated in the context of β1-aabs-induced DCM (with however a lower efficiency than therapies using neutralizing peptides)^30^. We previously showed that nebivolol treatment of coronary microvessels led to NO-dependent vasorelaxation^47^ while others documented a further protection of nebivolol administration against endothelial dysfunction though a net reduction in oxidative stress^48^. Altogether these studies suggest that the dual β1/β3-AR modulation, already demonstrated in previous studies for heart failure and myocardial infarction with Nebivolol^49^, could be extended to patients with β_1_-aabs-induced DCM.

In conclusion, we showed that UHF MRI allows the precocious detection of mouse myocardial remodeling induced by β1-aabs, at earlier time-points than anticipated based on previous reports using echocardiography. Importantly, this technology allowed us to identify a diastolic dysfunction occurring before the onset of systolic dysfunction in this autoimmune cardiac disease and to provide evidence supporting the therapeutic potential of drugs endowed with beta-3 AR agonistic activity for the treatment of β1-aabs-induced DCM.

## Methods

### Animals and immunization

All the experiments involving mice received the approval of the “Comité d’Ethique pour l′Expérimentation Animale de l′Université catholique de Louvain” (approval reference #2012/UCL/MD005) and were carried out according to national animal care regulations. This study conforms to the Guide for the Care and Use of Laboratory Animals published by the US National Institutes of Health (NIH Publication No. 85–23, revised 1996). Mice were housed under standard conditions with *ad libitum* access to water and chow.

#### C57BL/6J mice

A group of ten 8-weeks C57BL/6J male mice (Janvier, Paris, France) were monthly immunized with 200 μg of a synthetic peptide (Eurogentec, Seraing, Belgium) corresponding to the human and mouse β_1_AR-EC_II_ (residues 197–222: H-W-W-R-A-E-S-D-E-A-R-R-C-Y-N-D-P-K-C-C-D-F-V-T-N-R) through subcutaneous injection. The peptide was dissolved in 0.1 M Na_2_CO_3_/1% β-mercaptoethanol and emulsified in complete Freund’s adjuvant for the first injection and incomplete adjuvant for the followings in order to avoid excessive inflammatory response. Another group of 10 C57BL/6J male mice received only the vehicle and were used as controls. Mice were anaesthetized (150 mg/kg ketamine, 10 mg/kg xylazine, i.p.) prior each injection, allowing simultaneous collection of retro-orbital blood samples.

#### β3TG mice

Male mice harboring an α-myosin heavy chain promoter-driven human β3-AR transgene generated as described previously^50^, were used to produce heterozygous β3TG mice and wild-type littermate controls (n = 40); the original β3TG mouse line had been selected to exhibit a moderate overexpression of the transgene (matching the *ex vivo* myocardial response to a β3-AR agonist). Mice from β3TG and wild-type groups were randomly distributed in two subgroups and immunized according to the protocol described above, except that subcutaneous injections were performed under light anaesthesia with isoflurane 2–3% in oxygen for 3 minutes, and that no retro-orbital blood sample was taken at the same time to minimize animal stress. 12 mice per group were immunized with the peptide while 8 mice per group receive the vehicle and were used as controls.

#### Morphometric measurements

At the end of the study, after a follow-up of 28 weeks, animals were euthanized by cervical disclocation and their left ventricular weight and tibial length were measured. Hearts and sera were collected for analysis and stored at −80 °C.

### Autoantibody detection

To detect β1AR autoantibodies in mouse sera, the β_1_AR-EC_II_ synthetic peptide was used in an enzyme-linked immunosorbent assay (ELISA). Plates (Reacti-Bind, Thermo scientific, Rockford, IL) were coated overnight at room temperature with 1 μg/ml peptide (Eurogentec, Seraing, Belgium). Coating and blocking procedures were carried out using Ultrablock and Neptune buffers form AbD Serotec (Oxford, UK) according to the manufacturer’s instructions. Sera (diluted 1/2500) were then incubated overnight at 4 °C. After washing, specific hybridization was measured with a peroxidase-conjugated antimouse IgG antibody (dilution 1/10000) and addition of 3,3′,5,5′-tetramethylbenzidine from Calbiochem (Merck Chemicals, Nottingham, UK). The absorbance was determined at 450 nm in a microplate reader (Victor X4; Perkin Elmer, Waltham, MA).

### MRI measurements

#### Cardiac MRI (CMR) acquisition

Each animal was scanned at 10, 18 and 27 weeks of treatment on a 11.7T MRI scanner dedicated to small animal applications (Biospec, Bruker, Ettlingen, Germany). A quadrature ^1^H resonator was used for radiofrequency transmission (inner diameter = 72 mm, length = 6.6 cm) in conjunction with a surface receive-only coil array (length = 10.7 cm). Anaesthesia was induced with 3% isoflurane in oxygen, and then maintained with 0.5–2% isoflurane during the entire procedure, in order to remain within physiological heart rates (around 500 heartbeats/min). Animals were placed in prone position and monitored for electrocardiogram and respiration with neonatal electrodes wrapped around the paws and a pneumatic sensor placed under the animal. The body temperature was followed by using a rectal probe and regulated with a dedicated heating blanket. Cardiac scout images were obtained in the conventional planes with a tripilot sequence. Then an Intragate 2D cine Fast Low Angle Shot (FLASH) sequence was applied to acquire a stack of seven to eight 1-mm thick contiguous short-axis images covering the entire ventricles, perpendicular to the LV long-axis. Imaging parameters: repetition time: 5.83 ms; echo time: 1.45 ms; flip angle: 25°; field of view: 30.0 × 30.0; and matrix size: 256 × 256; resulting in an in-plane resolution of 0.12 × 0.12 mm^2^.

#### CMR Image analysis

The LV systolic function was assessed from the stack of short axis images by tracing epicardial and endocardial borders on Segment software (Medviso v1.8, Lund, Sweden). End-diastolic (EDV), end-systolic (ESV) and stroke volume (SV) were determined (μl). LV ejection fraction (EF, as %) and LV mass (mg) were subsequently deduced. Systolic and diastolic indexes were determined as previously described^41^,^42^. Briefly, we visually determined the end-diastolic phase, end-systolic phase and the phase at 30% of diastole, and traced the endocardial contours at the mid-ventricular level on the Osirix imaging software (v4.0; Pixmeo; Geneva, Switzerland). Then we calculated the systolic fractional area change (systolic index, as %) through the following formula: EDA-ESA/EDA, where EDA = end-diastolic area and ESA = end-sytolic area. The fractional area change during the first 30% of diastole (diastolic index, as %) was also calculated through the following formula: dA-ESA/EDA-ESA, where dA = area at 30% of diastole. All analyses were performed on a blinded basis.

### Cardiac histochemistry

At the end of the study hearts were excised and representative pieces of left ventricles were fixed in 4% formaldehyde after rinsing with saline. For analysis of cardiac fibrosis, 5-μm sections of paraffin embedded hearts were stained with picrosirius red. Stained sections were digitalized with a SCN400 slide scanner (Leica Biosystems, Wetzlar, Germany). Quantification was made with Tissue IA software (Leica Biosystems, Dublin, Ireland). Area occupied by interstitial fibrosis was expressed as a percentage of total myocardial area. To quantify transverse cardiomyocyte area and capillary density, cryosections were stained with Wheat germ agglutinin (WGA) as a membrane marker, and with biotinylated-Isolectine B4 as an endothelial marker. Mounted slides were observed with an Axioimager Z1/Apotome microscope equipped with a MRM camera (Zeiss, Germany). The data analysis was performed with Axiovision software (Zeiss, Germany). A minimum of 40 to 100 cells from 9 sections were measured in each heart. Other heart cryosections were incubated with antibodies raised against the monocyte marker CD11b and the lymphocytic marker CD45 (rat anti-CD11b and rat anti-CD45, both from BD Bioscience, San Jose, CA). Slides were scanned with a MIRAX Scanner (Zeiss, Germany) and analyzed with FRIDA software (Johns Hopkins University, Baltimore, MD). All analyses were performed on a blinded basis.

### Quantitative Polymerase Chain Reaction (qPCR)

Total RNA was isolated from heart tissues using TRI-reagent (Fermentas, Alost, Belgium) according to the manufacturer’s instructions. 1 μg total RNA was reverse transcribed with RevertAidTM M-MuLV Reverse Transcriptase (Thermofischer Scientific, Alost, Belgium) using hexamer primers. Resulting cDNA served as template for quantitative real-time PCR analysis using the following primers: *Adrb1* (Forward: 5′-GCTGATCTGGTCATGGGATT-3′, Reverse: 5′-AAGTCCAGAGCTCGCAGAAG-3′), *Adrb2* (Forward: 5′-TTCGAAAACCTATGGGAACG-3′, Reverse: 5′-GGGATCCTCACACAGCAGTT-3′), *Adrb3* (Forward: 5′-ACCAGAAGCCCTCAGCATCCCA-3′, Reverse: 5′-CACCCGCTTGTTTCAGGAGTCAC-3′), *Myh6* (Forward: 5′- GGGACATTGGTGCCAAGAAGA-3′, Reverse: 5′-ATTGTGGATTGGCCACAGCG-3′), *Myh7* (Forward: 5′-ACCAACCTGTCCAAGTTCCG-3′, Reverse: 5′-ACTCCTCATTCAGGCCCTTG-3′), *Nppa* (Forward: 5′TGATAGATGAAGGCAGGAAGCCGC-3′, Reverse: 5′-AGGATTGGAGCCCAGAGTGGACTAGG-3′), *Nppb* (Forward: 5′-GCCAGTCTCCAGAGC AATTC-3′, Reverse: 5′-AGCTGTCTCTGGGCCATTT-3′), *Gapdh* (Forward: 5′-TGCACCACCAACTGCTTAGC-3′, Reverse: 5′-GGATGCAGGGATGATGTTCT-3′). All samples were processed in triplicate reactions using Takyon for SYBR low Rox (Eurogentec, Seraing, Belgium) on the ViiA7 Real-Time PCR System (Applied Biosystems) using a fast cycling protocol. GAPDH was used as a reference endogenous gene to normalize the results. Results are shown as fold expression relative to untreated samples according to the ΔΔC_T_ method.

### Statistical Analysis

Data are expressed as mean ± SEM. Raw data were analyzed for normal distribution using the Shapiro-Wilk test. When normally distributed, unpaired Student *t* test was used to compare differences between two groups and one-way analysis of variance followed by Bonferroni post-hoc test was used to compare 3 or more groups. In the absence of normal distribution, the statistical significance of differences was tested with non-parametric tests (Mann-Whitney or Kruskall-Wallis followed by Dunn’s test). *P* < *0.05* was considered statistically significant. Statistical analyses were performed with GraphPad Prism 5.04 (San Diego, CA) and JMP 11.2.0 (SAS Institute, Cary, NC).

## Additional Information

**How to cite this article:** Vanhoutte, L. *et al*. MRI Assessment of Cardiomyopathy Induced by β1-Adrenoreceptor Autoantibodies and Protection Through β3-Adrenoreceptor Overexpression. *Sci. Rep.* **7**, 43951; doi: 10.1038/srep43951 (2017).

**Publisher's note:** Springer Nature remains neutral with regard to jurisdictional claims in published maps and institutional affiliations.

## Figures and Tables

**Table 1:** *In vivo* MRI parameters in mice at 10, 18 and 27 weeks and *ex vivo* data for LV mass and LV/TL after sacrifice at 28 weeks.

	10 weeks		18 weeks		27 weeks	
	CTL	AdrB1+	CTL	AdrB1+	CTL	AdrB1+
n	10	9	10	7	10	7
EDV (μl)	56,4 ± 1,6	64,2 ± 3,8	59,6 ± 1,5	69,1 ± 4,3*	57,2 ± 1,2	67,3 ± 2,5**
ESV (μl)	24,0 ± 2,5	22,9 ± 2,0	27,0 ± 13,8	32,0 ± 14,0	22,0 ± 1,5	29,0 ± 4**
SV (μl)	32,6 ± 2,3	41,4 ± 3,2*	29,0 ± 16,3	41,0 ± 21,0	35,3 ± 1,0	39,6 ± 2,6
EF (%)	57,7 ± 4,0	64,1 ± 2,7	51,0 ± 15,5	59 ± 7,0	61,8 ± 1,1	58,7 ± 2,4
LV mass MRI (mg)	74,1 ± 1,7	81,1 ± 2,1*	74,8 ± 1,4	83,9 ± 3,6*	75,9 ± 1,1	88,0 ± 2,0****
LV/TL MRI (mg/mm)	3,8 ± 0,1	4,2 ± 0,1*	3,9 ± 0,1	4,4 ± 0,2**	3,9 ± 0,07	4,6 ± 0,1***
ES septum thickness (mm)	1,5 ± 0,1	1,3 ± 0,1	1,2 ± 0,1	1,4 ± 0,1	1,4 ± 0,1	1,4 ± 0,1
Systolic index (%)	66,8 ± 1,5	67,8 ± 2,5	66,8 ± 1,9	63,2 ± 3,0	66,5 ± 1,9	64,8 ± 2,0
Diastolic index (%)	59,6 ± 1,9	57,2 ± 2,1	64,5 ± 2,6	53,5 ± 5,6	63,1 ± 4,3	51,4 ± 1,1*
LV mass *ex vivo* (mg)					97,9 ± 1,9	107,5 ± 2,7**
LV/TL *ex vivo* (mg/mm)					5,0 ± 0,1	5,6 ± 0,1**

n represents the number of animals in each group. EDV = end-diastolic volume; ESV = end-systolic volume; SV = stroke volume; EF = ejection fraction; LV = left ventricle; TL = tibial length; ES = end-systolic.*P ≤ 0.0.05; **P ≤ 0.0.01; ***P ≤ 0.0.001; ****P ≤ 0.0001 *vs* control.

**Table 2:** mRNA expressions in heart lysates after 28 weeks of treatment.

Gene	Control mice	Immunized mice	p-value
ADRB1	1.00 ± 0.07	0.98 ± 0.10	0,87 (ns)
ADRB2	1.00 ± 0.07	0.94 ± 0.07	0,60 (ns)
ADRB3	1.00 ± 0.30	2.02 ± 0.39	0,11 (ns)
MYH6	1.00 ± 0.34	1.56 ± 0,58	0,39 (ns)
MYH7	1.00 ± 0.31	0.64 ± 0,26	0,40 (ns)
ANP	1.00 ± 0.19	1.14 ± 0.24	0,65 (ns)
BNP	1.00 ± 0.11	1.83 ± 0.46	0,07 (ns)

Data are presented as mean ± SEM, mRNA expressions in β1-immunized mice (n = 7) are normalized to expression measured in control mice (n = 10).
